# Cobotic drilling assistant for orthopedic surgery using Gaussian process-based breakthrough detection

**DOI:** 10.1007/s11548-026-03679-z

**Published:** 2026-05-08

**Authors:** Lucas Gimeno, Nils Johnson, Tobias Stauffer, Quentin Lohmeyer, Mirko Meboldt

**Affiliations:** https://ror.org/05a28rw58 grid.5801.c 0000 0001 2156 2780 PDZ, ETH Zürich, Rämistrasse 101, 8092 Zürich, Zürich Switzerland

**Keywords:** Robotics, Human–robot collaboration, Orthopedic surgery, Fracture treatment

## Abstract

****Purpose**:**

We introduce a cobot assisted cortical bone drilling, as applied in femoral, or clavicular fractures. This requires high accuracy, as minor deviations can cause complications through soft tissue penetration (STP). Human–robot collaboration (HRC) combines the surgeon’s expertise with robotic stability and sensing accuracy. Thereby, the robot assistant passively guides the drill and prevents soft tissue penetration by detecting breakthroughs via integrated f/t and joint velocity sensors and stopping in place.

****Methods**:**

A robotic arm that passively guides the surgeon during drilling and actively stops upon breakthrough. The detection algorithm is online and model-free, instead relying on online Gaussian process (GP) regression to adapt to varying drilling conditions. The system was evaluated in a user study with an experimental group of N=16 and a control group of N=17 participants, whereby STP was measured using a depth camera.

****Results**:**

Femoral, ulnar, and generic bone samples were drilled with initially sharp 3.2 mm drill bits. Across all bone types, the experimental group outperformed the control group by 6.79 mm (*p*< 0.001) and undercut the reported STP for experienced surgeons, 5.1 mm by 2.54 mm (*p*< 0.02) for the generic bone case, despite the fact that only lay participants were tested in this study. Baseline algorithms such as thresholding were also outperformed by the proposed method.

****Conclusion**:**

These findings provide evidence toward the potential of HRC and model online breakthrough detection methods to increase safety in orthopedic drilling and represent a promising step toward future clinical implementation and surgical training applications.

**Supplementary Information:**

The online version contains supplementary material available at https://doi.org/10.1007/s11548-026-03679-z.

## Introduction

Cortical bone drilling is a common surgical procedure used in the treatment of bone fractures through open reduction [1].

Critical for the success of such operations is the precise insertion of screws inside the bone, requiring the drilling of pilot holes. This often leads to soft tissue penetration (STP), potentially causing various neurovascular injuries as reported in the literature [2–5]. Minimizing STP is challenging due to difficulties differentiating bone from soft tissue [6] and because of high human reaction times of around 0.2 seconds [7]. A representative quantification of these effects can be found in [8]. Experienced surgeons exhibited a mean penetration depth of 5.1 mm in the case of sharp and 21.1 mm when using blunt drill bits in normal bone and 5.4 mm (sharp), resp. 13.9 mm (blunt) in osteoporotic bone. The reported penetrations in [8] could have caused severe iatrogenic damage, leading to irreversible impairment or even lethal injuries, depending on the drilling site [2].

As full automation of this task is limited by task variability, safety concerns, regulatory constraints, and cost, we focus on solutions that retain surgeon control [9] in the following discussion of prior work.

**Table 1:** Comparison of existing plunge mitigation and breakthrough detection approaches in orthopedic drilling

Method	Penetration/accuracy	Main limitation
Mechanical drill stopper [12]	2.92±0.5 mm	Requires depth estimation
SmartDrill (dual motor) [10]	0.9±0.5 mm	High cost, feed rate and rpm tuning
Two-handed technique [11]	10.6±3.5 mm	Skill dependent
Bone layer classification [14]	84.2% acc.	Detection only
Kalman filter [18]	N/R	Calibration required, automated system
Acoustic event detection [20]	93.6% sens.	No plunge prevention
This work	2.65 ± 2.30 mm	Probabilistic detection

**Table 2:** Study parameters of existing plunge mitigation and breakthrough detection approaches in orthopedic drilling

Method	Drill bit size	Bone type
Mechanical drill stopper [12]	3.2 mm	Synthetic, osteoporotic femur
SmartDrill (dual motor) [10]	3.2 mm	Synthetic, normal bicortical block
Two-handed technique [11]	4.5 mm	Synthetic, normal bone tube
Bone layer classification [14]	3.5 mm	Bovine, normal femur
Kalman filter [18]	2.5 mm	Porcine, normal femur
Acoustic event detection [20]	3.2 mm	Human, normal hip bone
This work	3.2 mm	Synthetic, normal femur, ulna, bone tube

Related works for STP mitigation include controlled advancement or dual-motor drills [10], two-handed drilling techniques [11], and mechanical drill stoppers [12]. Breakthrough detection has additionally been studied using acoustic emission signals [13, 14], supervised learning of force or vibration profiles [15], and model-based force predictions. However, accurate modeling of bone drilling remains challenging due to complex drill bit geometry, cutting mechanics, and material-dependent properties [16]. Furthermore, methods based on force/velocity thresholds or Kalman filters rely on predefined parameters and task-specific calibration [17–19].

Mechanical drill stoppers report mean plunge depths of approximately 2.9 mm in synthetic, osteoporotic bone but require prior estimation of bone thickness [12]. Dual-motor drills have been reported to achieve 0.9±0.5 mm STP, albeit at extensively high costs (USD 48k–74k) [10], and require prior tuning of the feed rate and rotational speed of the drill for each case. Furthermore, the only systematic large-scale validation was performed on bicortical saw blocks at vertical drilling angles [10]. Acoustic and deep learning-based methods report sensitivities of 84.2–93.6%, but primarily focus on bone state recognition rather than explicit breakthrough detection or plunge depth reduction, and require prior data generation and training [13, 14, 20]. A comparative overview is provided in Table 1. The relevant study materials (drill bit size, bone type) are listed in Table 2.

To address the limitations in cost, autonomy, and generalization, this work presents an entirely different approach, based on model-free, online Gaussian process (GP)-based breakthrough detection on a low-cost, generic collaborative robot, passively guiding the surgeon and actively stopping upon breakthrough. The system requires neither preoperative imaging nor explicit drilling models. The usage of HRC in surgery is a growing trend [21–24], and therefore, finding additional use cases for existing hardware can reduce costs. Additionally, the assisted drilling trajectories could be recorded without external tracking systems and subsequently reused for further operating steps, such as the insertion of bone screws.

In this approach, the robot acts as an adaptive safety layer, intervening only upon detected breakthroughs. A GP-based algorithm learns the individual surgeon’s drilling behavior during cortical engagement and identifies loss of resistance through real-time deviation from the observed quasi-steady drilling state, without relying on explicit drilling models, prior data, fixed thresholds, or task-specific calibration. Conceptually, ML-based online breakthrough detection represents a new paradigm: increased adaptability and practical robustness across drilling conditions at the cost of analytical guarantees. Within a collaborative framework, this enables real-time compensation for human reaction times and provides an alternative foundation for future improvements in robustness and formal safety assurance.

With this work, we aim to validate the potential of this novel approach in terms of safety and practicability by answering the following questions:

- Can the proposed system significantly reduce STP in normal bone compared to manual drilling in a controlled experiment?
- Does the proposed breakthrough detection algorithm achieve state-of-the-art results, or at least similar results to baseline thresholding, adaptive thresholding, and derivative-based methods?
- What are the impacts on mental and physical load for the operator compared to manual drilling when using this system?

## Materials and methods

### Overview

For the proposed system to be effective, the human–machine interface (HMI), the robot’s control system, and the breakthrough detection algorithm have to be effectively coordinated. Furthermore, the experimental setup needs to closely mimic the use case and allow for precise tracking of the STP.

We rely on a simple HMI using the FR3 research Robot^1^, based on hand guidance and a foot pedal. These design choices are based on recent findings pertaining to practical usability [25]. The robot uses a Cartesian impedance controller running at 1 kHz, maintaining the required orientation and stopping upon a detected breakthrough. The detection itself is achieved via online GP regression. Resultant forces acting on the drill are estimated via integrated link-side torque sensors on the robot, while the drill velocity can be estimated through the robot joint velocities, as the drill is rigidly attached to the arm. Polyurethane synthetic bones widely used for surgical training^2^are used to simulate the drilling process. STP is then estimated with a depth camera. For implementation details and codebase, refer to the additional submission material.

### Breakthrough detection algorithm

A probabilistic, GP-based detection algorithm with an RBF kernel is employed to learn the drilling behavior model-free for cortical bone online. This strategy makes the model highly generalizable as it learns the surgeons cortical bone drilling behavior in real time. Note that this process begins anew every time a new hole is drilled—the model is not initialized on any prior data. By refitting the Gaussian process regularly, but more slowly than the breakthrough dynamics, user variance is captured, while breakthroughs are underfitted. The smoothness of the RBF Kernel is therefore ideal. When the measured drilling velocity overshoots the 95% confidence bound, as shown in Fig. 1 and the following heuristics are fulfilled, a breakthrough is detected. The drilled bone is assumed to be healthy. This facilitates comparison to other methods, as they predominantly cover normal bone types (see Introduction). This leads to two distinct drilling regimes in cortical bone and the medullary canal. In contrast, force and velocity patterns in osteoporotic or inhomogenously dense bone may deviate significantly from healthy bone. Additionally, the absence of rigid collisions at the interface between the bone marrow and the second cortical layer, could lead to piercingdominated breakthroughs instead of cutting-dominated ones. s

**Fig. 1:**
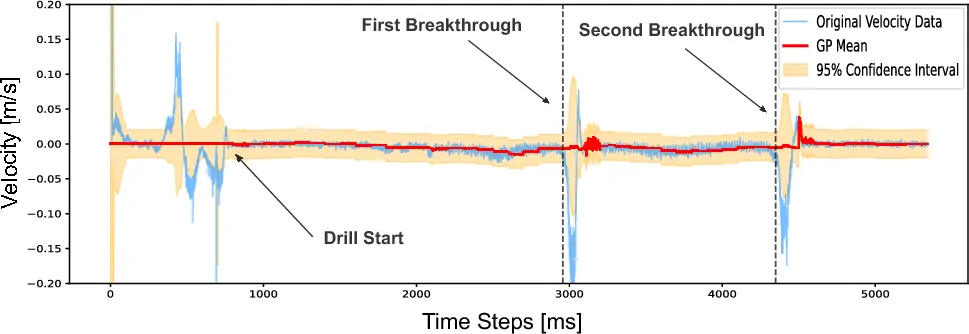
Velocity time series of a drilling process with corresponding online regression and confidence bounds

The frequency spectrum contribution from the drill operator is assumed to be in the range of 0–5 Hz, according to human reaction time [26], and significantly different from the contribution of the breakthrough dynamics in congruence with the healthy bone assumption. A fitting interval of 10 Hz follows, according to the Nyquist frequency criterion to avoid aliasing. Accordingly, physically informed hyperparameter bounds for the length scale were calculated based on[27], yielding a length scale around 1. This is expected due to steady-state and low frequencies dominating.

The variance is constrained to be between 0.01 ms$^{-1}$and 0.2 ms$^{-1}$, which is highly conservative for cortical bone drilling. This implies that the hyperparameter bounds allow sufficient flexibility for the GP to accommodate a wide range of drilling conditions. This was confirmed in prior experiments on generic bone and in the user study, where 99.99% of all values, including breakthroughs, fell within this interval.

Further, the drilling process is assumed to start directly from the top of the cortical bone layer. This avoids data contamination by positioning movements in free space, such as in the first 1000 time steps in Fig. 1, which are discarded by only considering values after the drill depth has reached 1 mm from the initial locking position. After the first breakthrough, a zero crossing of the velocity is expected before detecting the second breakthrough, avoiding temporary false positives (see velocity values outside the confidence interval in Fig. 1). The collision with the second cortical layer from within will ensure this condition is met. Furthermore, detections corresponding to positive resultant forces or velocities are discarded, as they are not physically possible. Finally, detections have to be triggered twice in a row to be considered, minimizing the effect of noisy outliers.

A pseudocode describing the algorithm can be found in code section 1.

**Algorithm 1:**
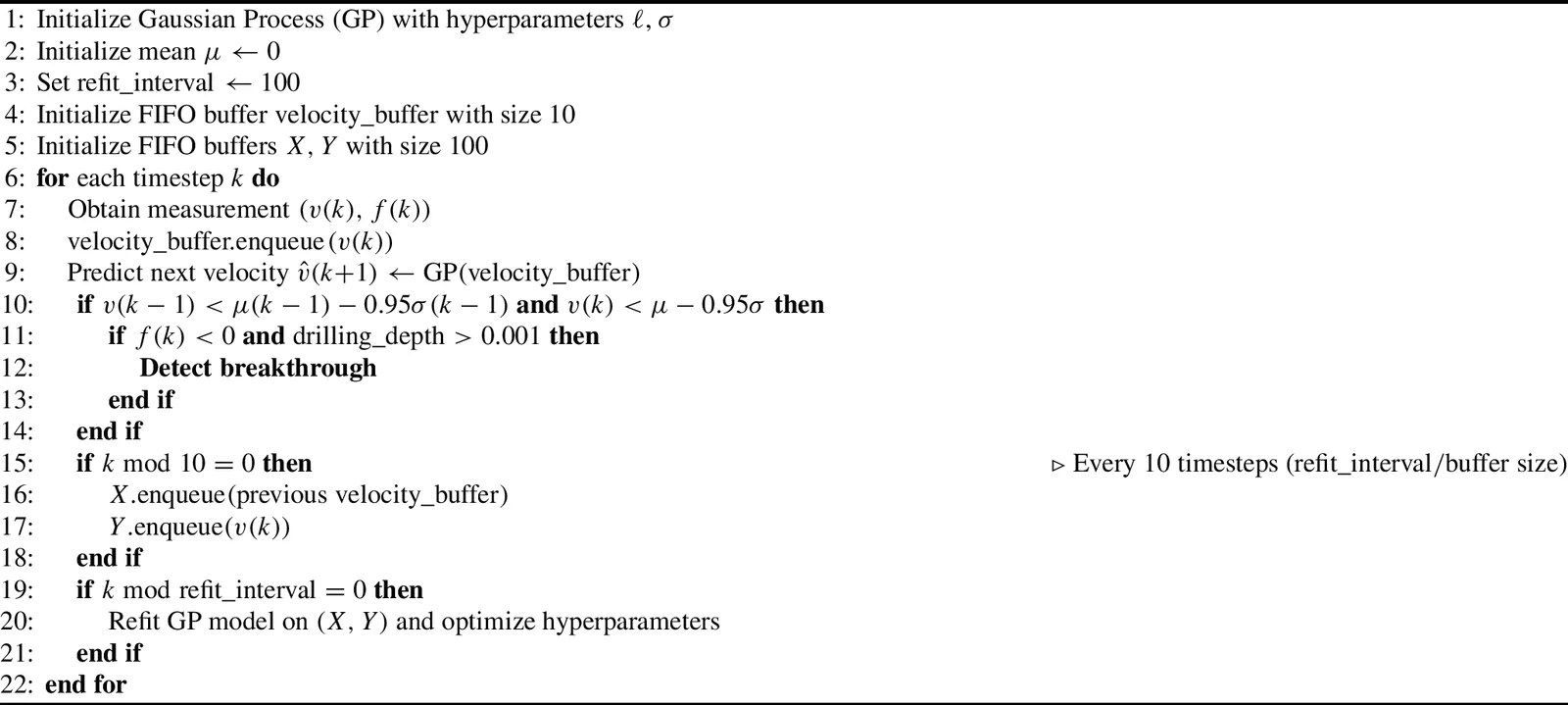
Online Gaussian Process Regression for Breakthrough Detection

### User study design

The experimental group (robot-assisted drilling, N=16) and the control group (manual drilling, N=17) were composed of persons with no prior surgical experience. Detailed information about the study demographics can be found in the supplementary material.

Participants drilled one hole per bone (generic, femur, ulna). Each time, a randomly generated reference direction (60, 70, 80, or 90 degrees w.r.t the longitudinal bone plane) was given. This forced drilling angle and geometric variations across experiments. RGBD data was gathered via depth camera for later STP estimation (see section Post-processing). The study setup showing a participant holding the drill at the specified angle is depicted in Fig. 2. For the manual group, the drill was simply dis-attached. The wall behind the bone mount increases the detection confidence of the depth camera, also visible in the picture. Lastly, the dimensions of the used drill bit (Synbone 3.2 mm) and a representative cross section of the used bones are shown. The drill bit size was chosen to be representative and as comparable as possible to the related literature values (see Introduction and Table User study design). At the start, a fresh drill bit was used, but was not changed until the end of the user study (including the manual group). This means that drill bit wear between approximately 0 and 100 usages significantly affected the drilling resistance across participants [28–30].

The robot is controlled using a foot pedal. First, the robot is released to be hand guided to the desired position and orientation. When the foot pedal is pressed again, the orientation is locked and the camera recording started. The surgeon then starts drilling and presses the foot pedal again when the process is finished. This unlocks the robot and stops the camera recording as well as all other data logs. At the end of the procedure, the participants are asked to fill out a NASA TLX^3^ questionnaire to assess mental and physical demand during the task, which would be crucial for practical application.

**Fig. 2:**
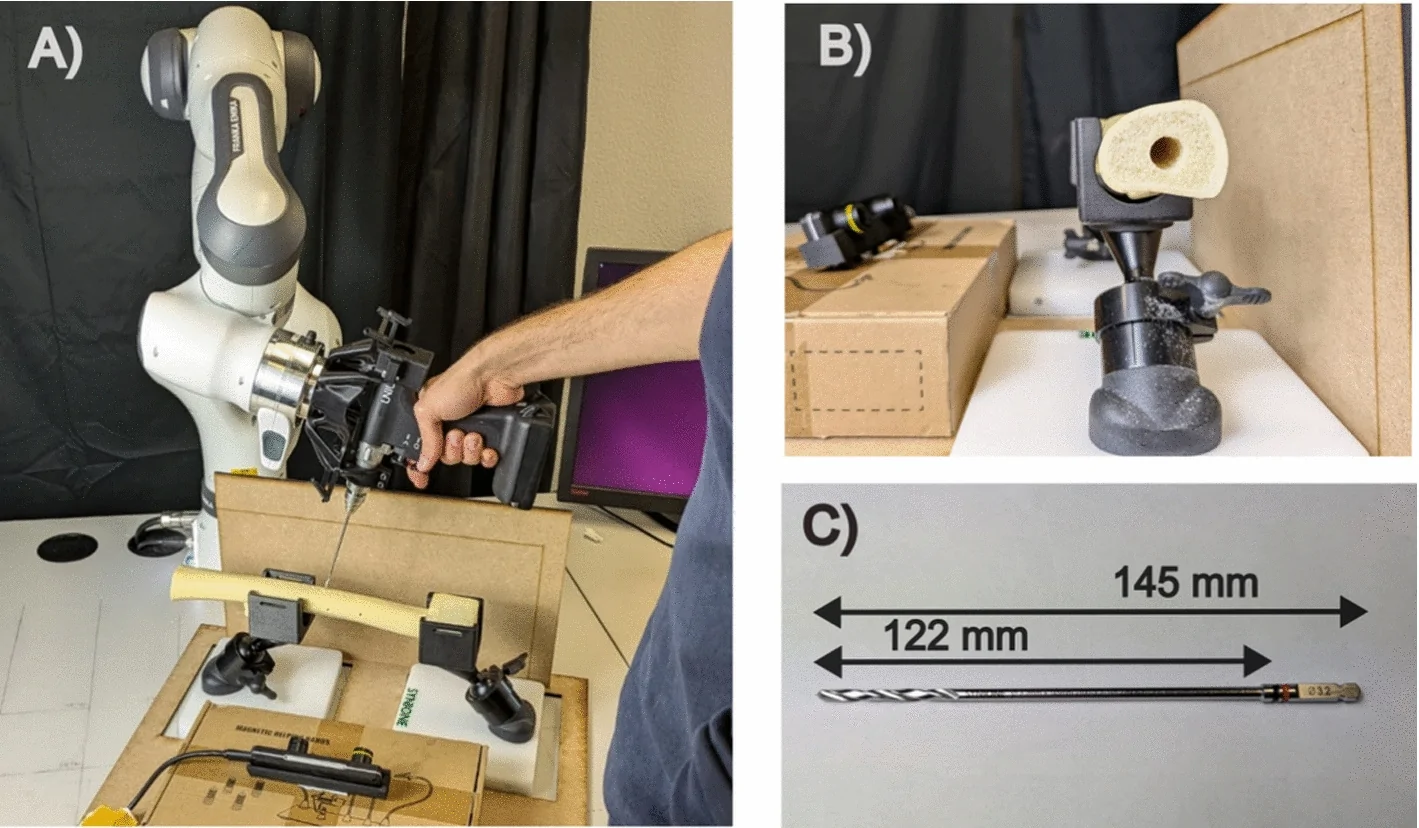
(**A**) Full setup (femur) with participant. (**B**) Side view of bone model in the user study setup (femoral bone model). (**C**) Used drill bit and drill bit dimensions

### Post-processing

To estimate STP, the frame corresponding to the highest drill overshoot and the real breakthrough event in the recording are retrieved by visual inspection. Then, the drill bit, drill tip, drill bit start, and the exit point in the bone are segmented manually. A line is fitted through the segmented points while constraining the start, end, and exit point of the drill to be as close as possible to the corresponding image ray given by the camera intrinsics and the pixel coordinates of the segmented points. Further, the distance between the start and end points of the drill bit is constrained to be 122 mm, corresponding to the real length of the segment (see Fig. 2). This allows a physically grounded estimate of the depth at the segmented locations and prevents NaN detections. Finally, the measurement uncertainty is estimated by fitting another line with PCA through the segmented point cloud, weighting each point by its detection confidence. The resulting STP uncertainty estimate is defined by the difference of 122 mm and the length between the start and end points of the drill bit projected on this PCA line. Figure 3 shows the confidence map of the depth image, the manual segmentation mask of the drill bit, start and end points, and exit point of the drill bit, as well as the line fit and the resulting segmented point cloud. To address cross-observer bias, the video frame extraction and segmentation were handled by different people. Two observers were assigned to do the segmentation separately, and the results were averaged.

### Handling of missing or incorrect data

In cases where the robotic system triggered too early, the data was imputed by entering the mean value for the manual drilling group at the corresponding metric, as it is assumed that the surgeon would just keep drilling to finish the operation. If the system did not trigger, we simply kept the final value. Both cases were still counted for the statistic, i.e., we report the total percentage of successful detections in the results section (see Results).

For both groups, the experiment was repeated if participants drilled into pre-existing holes or if they did not drill centrally, i.e., only drilled through cortical bone tangentially, instead of through the center of the bone. Data corresponding to such scenarios was discarded.

For the NASA TLX questionnaire, two values were imputed using mean imputation. For such a small amount of missing data, the benefit of keeping the rest of the questionnaire was weighed as higher.

**Fig. 3:**
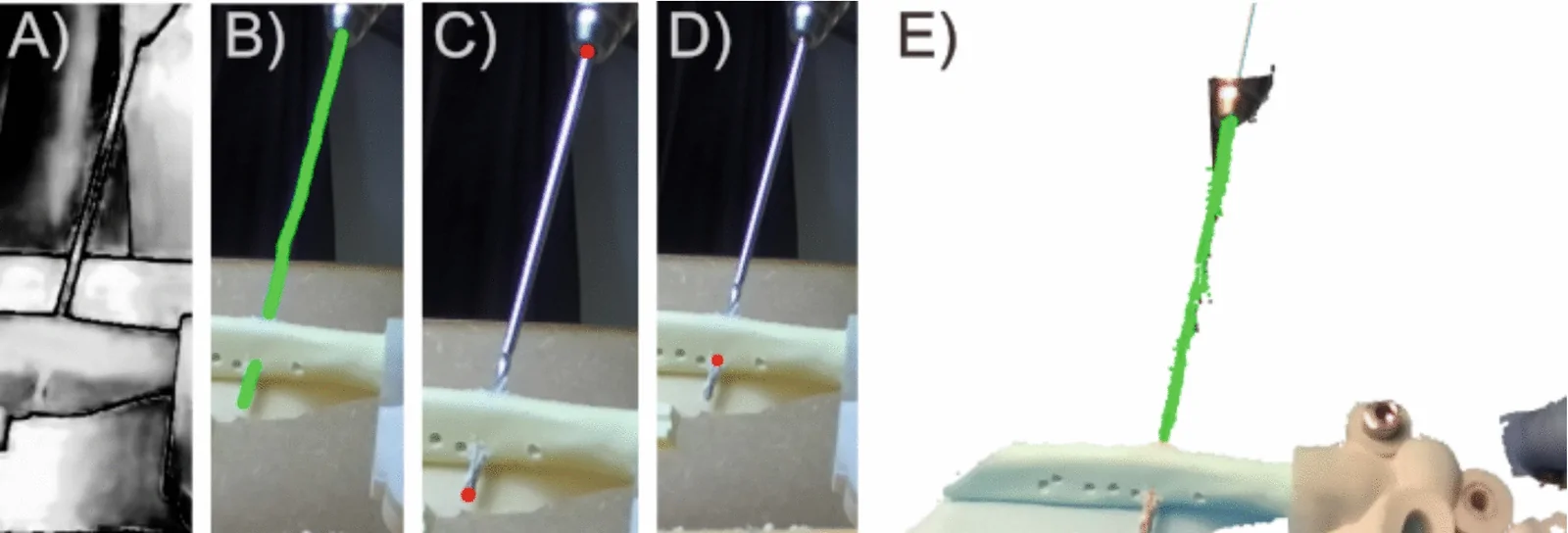
(**A**) Confidence of depth map of recorded image (white is more confident). (**B**) Manual segmentation of drill bit outside of drill. (**C**) Manual segmentation of start and end point of drill bit, which are constrained to have distance 122 mm. (**D**) Manual segmentation of exit point out of the bone (we measure STP from here). (**E**) Resulting 3D line fit with segmented high-confidence points in the scene

## Results

The following subsections report the detection performance of the proposed algorithm relative to baseline methods (Sect. Detection accuracy and latency), the resulting soft tissue penetration across bone types and in comparison with the literature (Sect. Soft tissue penetration), and the observed failure modes and their origins (Sect. Failure modes).

### Detection accuracy and latency

The overall success metrics are shown in Table 3, where the algorithm is further compared with simple baseline methods and a hand-tuned Kalman filter (KF). The average reaction time of the control system to a trigger was 10±10 milliseconds, thus significantly lower than the detection delay. The KF models the drilling velocity as a random walk with force-dependent process noise and detects sudden breakthroughs by thresholding the windowed normalized innovations using a Chi-squared test. The measurement noise was chosen according to the standard deviation of the velocity data, with the process noise modeled as dependent on force as $Q_{total} = Q_0 + f(k) * Q_f*dt$.

Force, velocity, and derivative force thresholding as well as adaptive thresholding via rolling mean were tuned on a 50/50 train–test split with fivefold cross-validation on the test set. Table 3 shows the performance on the test set (note that for our algorithm and the Kalman filter, we just display the performance on the full dataset). For implementation details, refer to the supplementary material and codebase. While simple thresholding already achieved a surprisingly high F1 score of 0.89, the GP-based breakthrough detection outperformed all others, achieving a F1 score of 0.95, while achieving the lowest detection latency of 160±70 milliseconds.

**Table 3:** Detection performance and detection delay comparison across methods. Rates are reported in percent rounded to two significant digits; time values are in milliseconds.

Method	F1	Accuracy	FP (%)	FN (%)	Median Δt (ms)	μ Δt (ms)	σ Δt (ms)
Gauss (Online GP)	0.95	0.90	6.3	4.2	150	160	70
Kalman Filter	0.79	0.64	13	20.0	177	209	119
$f_{\textrm{ext}}$ threshold	0.89	0.80	13	6.7	159	182	77
Velocity threshold	0.48	0.31	64	4.4	170	203	102
$\textrm{d}f_{\textrm{ext}}/\textrm{d}t$ threshold	0.62	0.44	49	6.7	168	184	65
$\textrm{d}f_{\textrm{ext}}/\textrm{d}t$ CI	0.59	0.42	56	2.2	170	179	81
$f_{\textrm{ext}}$ CI	0.75	0.60	31	8.9	139	172	92
Velocity CI	0.36	0.22	69	8.9	96	140	93

### Soft tissue penetration

Comparing the PCA-weighted approach to the constrained distance method yielded an average measurement uncertainty in the STP measurement of 1.28 mm with a standard deviation of 0.79 mm. The cross-observer uncertainty was found to be 0.58 mm

For the following statistical tests, the reported measurement uncertainty and cross-observer uncertainty has been added to all comparison groups pertaining to our own user study as additive noise, i.e., increasing the variance to account for measurement error.

Across all three bone types, STP was found to be 9.45±5.50 mm without and 2.65±2.30 mm with robotic assistance, classified per bone in Fig. 4. A Welch two-sample *t*-test (assuming unequal variance) comparing the robot-assisted and manual groups across all bones significantly rejects the null hypothesis of equal penetration depths (t=-7.6739, p<0.001).

Figure 4 presents the penetration depth distribution for the generic bone sample, enabling comparison with the literature values reported for experienced surgeons in [8], which used the same 3.2 mm drill bit, bone type, and drill. In that study, participants drilled three holes into a Synbone generic bone sample, and penetration depth was measured using a drill stopper, subtracting the known bone diameter from the protruding drill length. The study reported a mean soft tissue penetration depth of 5.10±3.02 mm for experienced surgeons. Our robotic assistance reduced the mean penetration depth by 48% and the standard deviation by 24% compared to experienced surgeons when using the same bone type, surgical drill, and drill bit.

A Welch two-sample *t*-test confirms this effect to be statistically significant, rejecting the null hypothesis of equal means with t=-2.5717 and p<0.02. The standardized effect size, computed as Cohen’s *d* using the pooled standard deviation, was d=0.96, indicating a large effect.

The statistical evaluation of the NASA TLX scores is reported in Table 4. The null hypothesis was rejected only for the performance metric, indicating that the robotic assistance did not impose additional workload on the users and helped them achieve the task at hand.

**Fig. 4:**
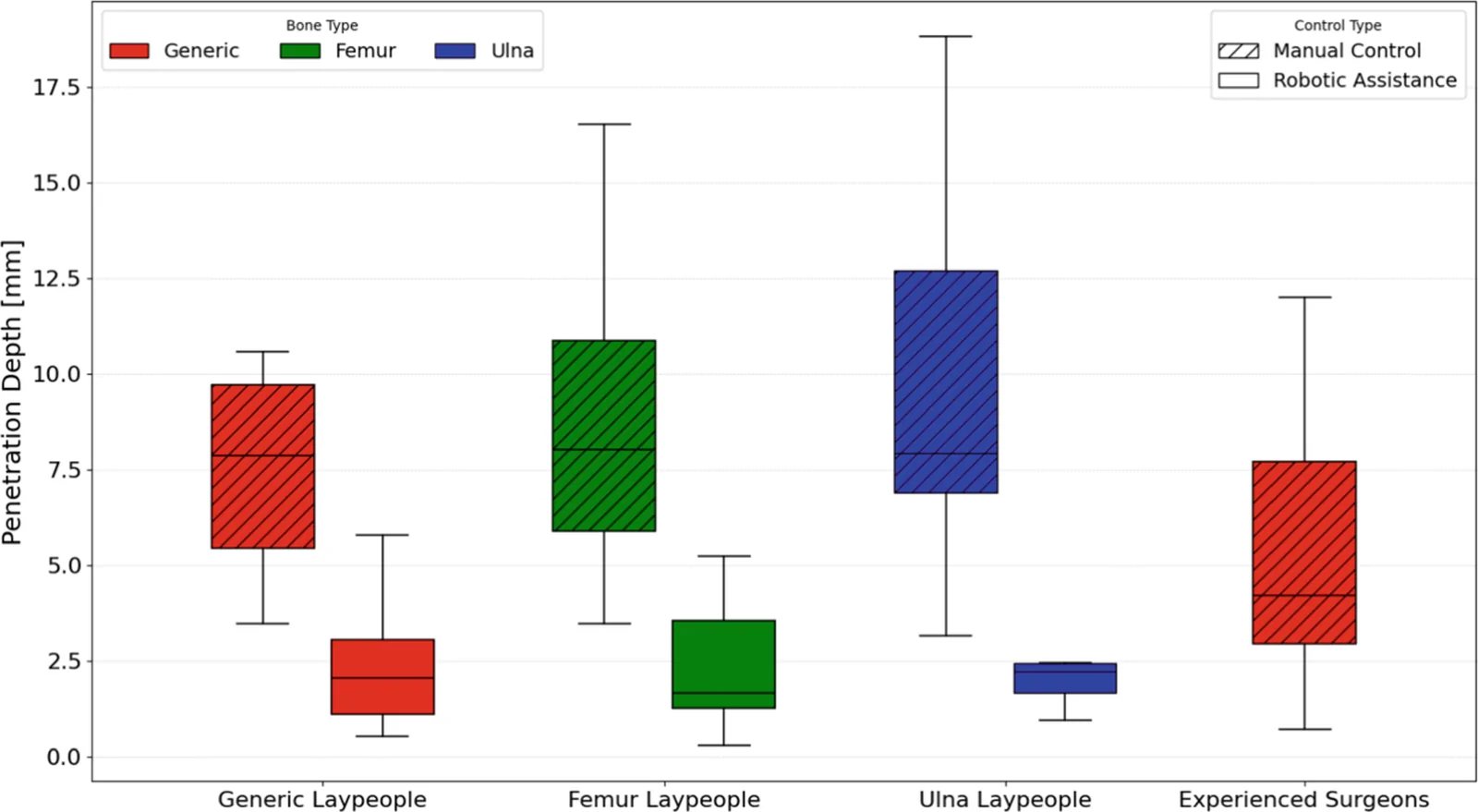
Comparison of soft tissue penetration in generic, femoral, and ulnar bone

### Failure modes

Table 3 reports a false positive rate of 6% and a false negative rate of 4%, yielding a total error rate of 10%. False positives were primarily caused by noisy outliers misclassified as breakthroughs, while false negatives occurred when the Gaussian process adapted too rapidly to the breakthrough regime. Notably, none of the errors originated from the heuristics, suggesting that the assumptions of distinct frequency contributions between medium change and operator movement, as well as rigid collision at the second cortical bone layer, are reasonable for healthy bone. The femur samples exhibited the highest failure rate at 19% (1 FN, 2 FP), compared to 5% for both the generic (1 FN) and ulnar (1 FP) samples. Despite the small sample size, this is consistent with the greater material complexity of the femur bones, which were denser and contained Spongiosa unlike the hollow generic and ulnar samples. This points to material variations as a potential failure mode, connecting to the broader limitation of pathological bone discussed in Sect. Discussion.

**Table 4:** Comparison of NASA TLX workload dimensions between manual and robot groups.

Group	Mental Dem.	Phys. Dem.	Temp. Dem.	Perf.	Effort	Frustr.
Manual	4.76 ± 4.15	4.41 ± 3.74	4.47 ± 3.2	7.71 ± 3.64	4.94 ± 4.10	4.06 ± 4.02
Robot	6.94 ± 3.42	5.00 ± 3.69	3.81 ± 2.29	5.00 ± 2.45	5.75 ± 3.86	4.94 ± 3.79
*p-value* $^{1}$	0.11	0.65	0.5	0.018	0.56	0.52

$$^{1}$$ $$H_0$$: $$\mu _{Manual} = \mu _{Robot}$$

## Discussion

### Experimental validation

The drilling assistant demonstrated promising results, significantly reducing STP between the control and experimental groups. The proposed algorithm detected the second breakthrough correctly in the vast majority of cases, outperforming force, velocity, and derivative force thresholding, as well as rolling mean and KF-based algorithms in both accuracy and speed. The reported sensitivity of 93.49% is comparable with learning- and model-based methods using acoustic signals, which report values between 84 [14] and 93% [20]. The detection latency of 160±70 ms suggests that the majority of the STP reduction stems from the fast reaction time of the robot control to the breakthrough signal (10±10 ms), rather than from a detection speed advantage over human reaction time. Nevertheless, the robot-assisted group achieved STP values that significantly undercut the 5.1 mm mean reported for experienced surgeons [8]. Although these comparison data originate from a separate study, the bone samples, surgical drill, and drill bits were *identical* across both experiments, minimizing material and procedural heterogeneity. Since the robotic condition was performed by laypersons, experienced surgeons would be expected to perform at least as well with the same assistant. If anything, the true benefit may be underestimated.

Furthermore, the observed penetration depths for the femoral, ulnar, and generic bone types lie closely together (see Fig. 4), despite variation in bone geometry and drilling angle across experimental runs. In combination, these findings confirm that the presented system can account for differences in operator behavior and bone geometry in the tested conditions.

Desirable practical usability is confirmed by the NASA TLX results. A further potential benefit of the cobotic solution is repeatability—the drilling path recorded during the procedure could assist in subsequent screw insertion, improving both alignment and speed.

### Limitations

A remaining concern is the sensitivity of the method to variations in drill conditions, such as drill bit geometry, sharpness, and diameter. Unlike approaches that rely on pre-trained models with fixed feature distributions, the proposed GP learns online from the initial phase of each drilling session. As drilling begins, the model incrementally adapts to the current force and velocity dynamics through regular refitting of the mean and uncertainty, and joint hyperparameter optimization within bounds at each step. Condition-dependent characteristics of the velocity profile are therefore absorbed into the model, rendering the detection principle robust to constant or slowly varying offsets—such as those introduced by drill bit wear or geometry changes—provided the fundamental structure of the breakthrough event is preserved.

This robustness is supported by the nature of the confounding factors. Drill bit wear, sharpness loss, and diameter increases all produce effects that are approximately constant during cortical bone drilling [28, 29, 31–33]: Wear uniformly elevates drilling forces across conditions [29, 30], larger diameters increase axial friction proportionally [31–33], and tool angle influences can be captured through a lumped parameter in the force model [34–36]. With approximately 100 drill bit usages across this study, considerable sharpness variation is expected to have been present, providing implicit coverage of wear-related effects. Nevertheless, some caution is warranted. Reporting standards across the drilling literature are heterogeneous, making direct quantitative comparisons across studies difficult, and the degree to which these findings transfer to substantially different drill bit configurations has not been systematically established. Furthermore, interactions between changing drilling forces and operator behavior—for instance, compensatory changes in applied feed rate or grip—could qualitatively alter the velocity profile in ways that a constant offset argument does not fully capture. Whether the GP’s online adaptation remains sufficient under such conditions, or whether explicit recalibration would be needed, remains an open question and a direction for future experimental validation.

The most significant limitation concerns pathological bone features and anisotropy. The synthetic bones used in the user study have approximately uniform cortical density, whereas low or highly variable bone densities present greater detection challenges: The velocity zero crossing heuristics (see Breakthrough detection algorithm) may fail, and the frequency contributions from the medium transition would overlap more closely with those of operator movement. Inhomogeneities could further introduce time- and depth-varying changes to the drilling dynamics and potentially violate the rigid collision assumption at the cortical interface. Crucially, the online GP adaptation that provides robustness to condition-dependent offsets in healthy bone cannot be expected to generalize to such qualitatively different dynamics. Quantifying these effects and developing mitigation strategies remains underexplored, presenting a persistent research gap.

## Conclusion and outlook

The user study demonstrated that the proposed robot-assisted drilling approach significantly reduces soft tissue penetration in orthopedic drilling procedures. Beyond the experimental results, the work contributes a novel human–robot collaboration framework centered on intraoperative safety, a public dataset to support further research, and a systematic comparison with existing detection methods.

The results are encouraging, yet several directions remain open. Naturally, practical aspects, such as workspace positioning, singularity avoidance, and instrument handling, remain directions for future improvement. Clinically, the next step would be to involve clinical professionals, both to benchmark the system directly against expert performance and to assess its behavior in realistic surgical environments. Controlled experiments with real bone and explicit variation of drill bit configurations and wear would further elaborate the robustness of the approach under clinically relevant conditions to confirm its potential generalization properties.

A separate and underexplored challenge is soft tissue penetration mitigation in pathological and osteoporotic bone. Existing techniques, including the present one, have been developed and validated primarily on healthy specimens, and the detection dynamics in low-density or highly inhomogeneous tissue are not well understood. Extending the current framework to such cases—for instance through revised heuristics suited to osteoporotic bone—represents a clinically meaningful research direction.

Finally, the thermal implications of robot-assisted drilling warrant further investigation. Thermally induced osteonecrosis remains a persistent concern in bone drilling, and the influence of the control strategies discussed here on heat generation has not yet been characterized.

## Declaration of generative AI and AI-assisted technologies in the writing process

During the preparation of this work, the author(s) used ChatGPT-5 and Claude Sonnet 4.6 in order to improve the clarity of the writing. After using this tool/service, the author(s) reviewed and edited the content as needed and take(s) full responsibility for the content of the publication.

## Supplementary information

We provide our participant introduction protocol, implementation details of the hardware, links to our public code repositories (data and post-process^4^, baseline comparison^5^, controller^6^, logger and detector^7^), the consent form, and a video of the procedure in the supplementary material. Additional explanations and access to additional data will be granted upon request.

## Supplementary Information

Below is the link to the electronic supplementary material.

Supplementary file 1 (mp4 9792 KB)

Supplementary file 2 (pdf 144 KB)

## Footnotes

1 FR3.

2 https://www.synbone.com/products/bone-models/.

3 NASA TLX Questionnaire.

4 https://github.com/LucasG2001/zed_breakthrough_detection.

5 https://github.com/LucasG2001/baseline_comparison.

6 https://github.com/njohnson433/drill_control.

7 https://github.com/njohnson433/drill_control_logger.

## Declarations

### Code availability

All code and data is shared via GitHub.
