# Supplementary material for: Cobotic drilling assistant for orthopedic surgery using Gaussian process-based breakthrough detection
Source: Int J Comput Assist Radiol Surg. 2026 May 8;21(6):1161–70. doi: 10.1007/s11548-026-03679-z (PMC13744559; doi:10.1007/s11548-026-03679-z)
Supplement: Supplementary file 1 [file 11548_2026_3679_MOESM2_ESM.pdf]

Demographics

Of the 33 total participants 88\% were men, 12\% were women and participants had a background mostly in engineering sciences. 9\% were left-handed and 91\% right-handed. The mean age was 27 years, with a standard deviation of 4 years. Each participant was instructed orally according to our protocol in the supplementary material and signed an informed consent form, giving consent to the collection and publication of the data presented here.

Computation Hardware

The Gaussian Process (GP) regression is based on the GPy Python package (https://gpy.readthedocs.io/en/deploy/). Finally, we use a ZED 2 Mini stereo camera (https://www.stereolabs.com/en-ch/store/products/zed-mini) to estimate STP, recording video at 30 fps with a resolution of 720p. The controller was executed on a dedicated workstation (AMD Ryzen 7 8700G, 32 GB RAM, Ubuntu 22.04.5 LTS, NVIDIA GeForce RTX 4090 with 24 GB VRAM), while the camera capture loop ran on a separate PC (AMD Ryzen 7 3700X, 16 GB RAM, Ubuntu 22.04.5 LTS, NVIDIA GeForce RTX 2080 with 8 GB VRAM). Communication between the robot, camera, and the workstations is facilitated using the ROS 2 middleware (https://docs.ros.org/en/humble/index.html).

Thresholding Parameters

"**velocity**": -0.03236301136164924 m/s

"**f_ext**": -3.2816402362456305 N

"**dt_f_ext**": -5.57004572246969 N/s

"velocity_ci"

"window": 800,

"ci_level": 99.9,

"f_ext_ci"

"window": 1000,

"ci_level": 99.9,

"dt_f_ext_ci"

"window": 400,

"ci_level": 99.9,

Kalman Filter Implementation

1. Problem Formulation

We aim to detect sudden changes in drilling velocity (breakthroughs) in real time. Velocity is measured along the drilling direction (velocity_desired) at 1 kHz. Force along the drilling axis (f_ext_desired) is available but may be noisy, and its causal effect on velocity is not explicitly modeled.

The detection problem is formulated as a sequential changepoint detection task: identify when the observed velocity deviates significantly from its nominal behavior.

---

2. Kalman Filter Model

Let v_k denote the measured velocity at time step k. The nominal pre-breakthrough dynamics are modeled as a random walk:

State equation:

$$x\_k = x\_\{k-1\} + w\_k, \quad w\_k \sim N(0, Q\_base + Q\_force * |f\_\{k-1\}|)$$

Measurement equation:

$$y\_k = x\_k + v\_k, \quad v\_k \sim N(0, R)$$

where:

- x_k is the latent state representing the expected velocity.
- y_k is the observed velocity (velocity_desired).
- w_k is the process noise, including a component that scales with the measured drilling force f_{k-1}.
- v_k is the measurement noise with variance R = σ_v^2.

- Q_base is the baseline process noise.
- Q_force is the additional process noise proportional to the force magnitude.

The Kalman filter performs the standard prediction and update steps:

Prediction step:

$$\hat{x}\_\{k|k-1\} = \hat{x}\_\{k-1|k-1\}$$
$$P\_\{k|k-1\} = P\_\{k-1|k-1\} + Q\_base + Q\_force * |f\_\{k-1\}|$$

Update step:

$$\nu\_k = y\_k - \hat{x}\_\{k|k-1\} \quad (innovation)$$
$$S\_k = P\_\{k|k-1\} + R$$
$$K\_k = P\_\{k|k-1\} / S\_k$$
$$\hat{x}\_\{k|k\} = \hat{x}\_\{k|k-1\} + K\_k * \nu\_k$$
$$P\_\{k|k\} = (1 - K\_k) * P\_\{k|k-1\}$$

where v_k is the innovation, S_k its variance, K_k the Kalman gain, and P_{k|k} the updated state covariance.

---

3. Changepoint Detection

Normalized innovations are computed as:

$r\_k = \nu\_k / \sqrt{S\_k}, \quad r\_k^2 \sim \chi^2{}\_1$ under the null hypothesis of normal drilling

To detect sudden deviations over a short temporal window, we define a windowed χ^2 statistic:

$$S\_k\^(W) = \sum\_\{i = k - W + 1\}\^\{k\}\, r\_i^2$$

where W is the number of samples in the detection window (typically 20–40 ms). Under the null hypothesis:

$$S\_k\^(W) \sim \chi^2\_W$$

A breakthrough (changepoint) is declared whenever:

$$S\_k\^(W) > \chi^2\_\{W, PFA\}$$

where χ^2_{W,PFA} is the PFA quantile of the χ^2_W distribution, and PFA is the user-specified probability of false alarm.

---

4. Tunable Parameters

Parameter	Description	Used Value / tested Range
Q_base	Baseline process noise	**1e-7** – 1e-5
Q_force	Force-dependent process noise	**1e-6** – 5e-6
R	Measurement noise variance	0.002^2
W	Window size for χ^2 detection	20–40 (**30**) samples (20–40 ms)
PFA	Desired false alarm probability	0.995–**0.999**
LEAK (optional)	Leaky integration for smoother predictions	**0**–0.02

Notes:

- Q_force inflates uncertainty during high-force periods without biasing the mean prediction.
- The window size W and PFA trade off detection latency versus false alarm rate.
- The filter is initialized using the mean of the first ~50 ms of velocity and a high covariance to allow rapid adaptation.

---

5. Implementation Notes

- Force is not integrated into the velocity prediction to prevent drift.
- The Kalman filter produces whitened residuals, which feed directly into the windowed χ^2 detector.
- The detector triggers when the windowed residual energy statistically exceeds the threshold, ensuring rapid detection (<150 ms) of velocity breakthroughs while remaining robust to noise.
